# A psychological intervention for suicide applied to non-affective psychosis: the CARMS (Cognitive AppRoaches to coMbatting Suicidality) randomised controlled trial protocol

**DOI:** 10.1186/s12888-020-02697-8

**Published:** 2020-06-16

**Authors:** Patricia A. Gooding, Daniel Pratt, Yvonne Awenat, Richard Drake, Rachel Elliott, Richard Emsley, Charlotte Huggett, Steven Jones, Navneet Kapur, Fiona Lobban, Sarah Peters, Gillian Haddock

**Affiliations:** 1 Division of Psychology and Mental Health, School of Health Sciences, Manchester Academic Health Sciences Centre, University of Manchester, Coupland Building 1, Oxford Road, Manchester, M13 9PL UK; 2 Greater Manchester Mental Health NHS Trust (formerly Manchester Mental Health and Social Care Trust), Manchester, UK; 3 Manchester Centre for Health Economics, School of Health Sciences, Manchester Academic Health Sciences Centre, University of Manchester, Manchester, UK; 4 Institute of Psychiatry, Psychology & Neuroscience, Kings College London, London, UK; 5 Lancashire Care NHS Foundation Trust, Lancashire, UK; 6 University of Lancaster, Lancaster, UK

**Keywords:** Suicide, Suicidal thoughts and behaviours, Psychological interventions, Cognitive therapy, Psychosis, Schizophrenia, Psychological suicide mechanisms, Randomised controlled trial

## Abstract

**Background:**

Suicide is a leading cause of death globally. Suicide deaths are elevated in those experiencing severe mental health problems, including schizophrenia. Psychological talking therapies are a potentially effective means of alleviating suicidal thoughts, plans, and attempts. However, talking therapies need to i) focus on suicidal experiences directly and explicitly, and ii) be based on testable psychological mechanisms. The Cognitive AppRoaches to coMbatting Suicidality (CARMS) project is a Randomised Controlled Trial (RCT) which aims to investigate both the efficacy and the underlying mechanisms of a psychological talking therapy for people who have been recently suicidal and have non-affective psychosis.

**Methods:**

The CARMS trial is a two-armed single-blind RCT comparing a psychological talking therapy (Cognitive Behavioural Suicide Prevention for psychosis [CBSPp]) plus Treatment As Usual (TAU) with TAU alone. There are primary and secondary suicidality outcome variables, plus mechanistic, clinical, and health economic outcomes measured over time. The primary outcome is a measure of suicidal ideation at 6 months after baseline. The target sample size is 250, with approximately 125 randomised to each arm of the trial, and an assumption of up to 25% attrition. Hence, the overall recruitment target is up to 333. An intention to treat analysis will be used with primary stratification based on National Health Service (NHS) recruitment site and antidepressant prescription medication. Recruitment will be from NHS mental health services in the North West of England, UK. Participants must be 18 or over; be under the care of mental health services; have mental health problems which meet ICD-10 non-affective psychosis criteria; and have experienced self-reported suicidal thoughts, plans, and/or attempts in the 3 months prior to recruitment. Nested qualitative work will investigate the pathways to suicidality, experiences of the therapy, and identify potential implementation challenges beyond a trial setting as perceived by numerous stake-holders.

**Discussion:**

This trial has important implications for countering suicidal experiences for people with psychosis. It will provide definitive evidence about the efficacy of the CBSPp therapy; the psychological mechanisms which lead to suicidal experiences; and provide an understanding of what is required to implement the intervention into services should it be efficacious.

**Trial registration:**

ClinicalTrials.gov (NCT03114917), 14th April 2017. ISRCTN (reference ISRCTN17776666 10.1186/ISRCTN17776666); 5th June 2017). Registration was recorded prior to participant recruitment commencing.

## Background

Suicidal thoughts, plans, and fatalities are of substantial concern worldwide. Approximately 800,000 lives are lost to suicide, globally, every year, which equates to one person dying by suicide every 40 s [1]. Estimates indicate that approximately 48,344 people died by suicide in the USA in 2018, which is 14.2 deaths per 100,000 population [2]. In the UK, for 2018 registrations, there were 6507 suicide fatalities, that is, 11.2 deaths per 100,000 population [3]. Suicide deaths increased in 2018 in the UK compared to 2013 figures [4]. In the US, the suicide death rate has also been increasing over time [5]. Suicide attempts are far more prevalent than suicide deaths, and suicide thoughts are more frequent than attempts. For example, as collected by the Adult Psychiatric Morbidity Survey, in 2014, 21.6% of British white people in England, UK, had suicidal thoughts whereas 6.9% had attempted suicide [6], and 0.01% had died by suicide that year. There were 1.4 million suicide attempts in 2017 in the USA [2] and 47,000 suicide deaths [7].

There is sound evidence that suicidal thoughts, plans, attempts and deaths are significantly elevated in those with severe mental health problems [8]. For example, mental health problems in those experiencing non-affective psychoses, or recent onset psychosis, have been associated with one of the highest, relatively unchanging, suicide mortality rates across the decades [9–12]. Whilst it is vital to focus on preventing deaths by suicide it is equally important to focus on reducing suicidal thoughts, plans and acts in people with severe mental health problems because they are both common and highly distressing for individuals and their families. For example, up to 50% of people with non-affective psychoses will experience suicidal thoughts and/or suicide attempts [10, 13]. Furthermore, suicidal thoughts and acts are associated with immense psychological distress [14] and can be strong predictors of suicide fatalities [15]. For instance, as reported by a recent meta-analysis, people with schizophrenia who also had thoughts of suicide were six times more likely to die by suicide compared to a 1.5 fold increase in those experiencing depression [16]. Hence, it is important that interventions which combat suicidal thoughts and acts are designed to attenuate a range of suicidal experiences, such as thoughts, urges, plans and attempts, in people with severe mental health problems such as psychosis.

A small number of meta-analytic systematic reviews have examined the extent to which psychological and psychosocial interventions reduce self-harm and suicidal experiences in those with mental health problems [17–21]. For example, two reported that psychological interventions were effective as long as they targeted suicidality (i.e., suicidal thoughts, behaviours and acts) or psychological precursors of suicidal experiences (e.g., hopelessness) rather than symptoms reflecting a psychiatric diagnosis [18, 19]. One of these reviews specifically evaluated the effects of cognitive-behavioural types of therapies (CBT) on suicide thoughts and behaviours [19]. Findings showed that cognitive behavioural therapies, including dialectical behavioural therapy, reduced suicidal thoughts and behaviours.

An issue with many studies included in such reviews is that few tested the effectiveness of a psychological talking therapy which, first of all explicitly focused on suicidal thoughts and acts, and second, were based on empirically supported recent psychological models which attempt to delineate the psychological pathways underpinning suicidal thoughts and behaviours. The Schematic Appraisal Model of Suicide (SAMS) [22] is one such recent psychological model of suicide which was developed from the influential Cry of Pain model [23] but also resonates with other contemporary psychological models of suicide, such as the Integrated Motivational Volitional Model [24–26]. These contemporary models converge, at least to some extent, in highlighting the importance of perceptions of emotional dysregulation, lack of social support and a perceived inability to solve inter-personal problems together with experiences of feeling defeated, trapped and hopelessness in pathways to suicidal thoughts and acts. It is important to understand mechanisms underlying suicidal experiences which are transdiagnostic and appear to be activated or triggered regardless of specific mental health problems or psychiatric diagnoses but to also recognise that specific symptoms associated with mental health problems, such as hallucinations, delusions, and hyperarousal may amplify suicidal thoughts, plans and acts [27–29].

Psychological therapies are optimally efficacious and effective if they focus on evidence-based, theoretically derived, psychological mechanisms thought to underlie suicidal thoughts and behaviours, and if as part of the therapy, they address such suicidal experiences explicitly [19]. Some of the cognitive behavioural therapies which have been evaluated may not always have been explicitly focused in this way. One psychological intervention approach which has focussed on ameliorating suicidal thoughts and acts is Cognitive Behavioural Suicide Prevention (CBSP) [14, 19, 30]. CBSP is based on the SAMS [22]. It was developed by the current authors and colleagues [14, 30, 31] as a psychological intervention designed to target suicidal thoughts, urges, plans and acts by focusing on perceptions of poor emotional regulation, social isolation, and difficulties with interpersonal problem solving. As these negative appraisals are posited to give rise to perceptions of being defeated, trapped and hopeless over time [32], and, in accord with a large body of evidence showing that these perceptions are central in the pathways to suicidal experiences [28, 32–42], CBSP also targets perceptions of defeat, entrapment and hopelessness. Hence, CBSP is unique in i. being grounded in a testable, evidence based psychological model of suicidal thoughts and acts, and ii. having an explicit and central focus on suicidal thoughts and behaviours.

Three pilot Randomised Controlled Trials (RCTs) have shown that the CBSP intervention is feasible and acceptable when adapted for use across a range of settings, and have suggested that it can be efficacious in reducing suicidal thoughts and acts. The first found reductions in measures of suicidal ideation and suicidal probability in people experiencing non-affective psychosis who lived in the community [30, 43]. The second, found that suicidal behaviours were less frequent in male prisoners following the CBSP intervention [31]. The third, found that this intervention was feasible, acceptable, and safe, and suggested that it may be cost-effective when used in psychiatric in-patient settings [44]. This means that the evidence from pilot RCTs across three diverse settings indicated that the CBSP suicide focussed therapy has the potential to counter suicidal experiences.

In order to develop this work further it is important to test the efficacy of CBSP in a larger, definitive, RCT in people with severe mental health problems who are vulnerable to suicidal experiences. It is also important to test the extent to which the underlying psychological mechanisms, on which CBSP is based are supported when applied to people with non-affective psychosis. Therefore, we propose to test CBSP adapted for people with non-affective psychosis (CBSPp) in an RCT, with two arms i. the CBSPp intervention plus treatment as usual (CBSPp plus TAU) and ii. treatment as usual (TAU) across three time points of baseline, 6 months follow-up (at therapy cessation), and 12 months follow-up. Our CARMS RCT is novel because i. it is founded on a testable psychological theory, ii. it is based on pilot work in diverse settings of a suicide focused cognitive therapy, and iii. unlike many of the studies included in recent meta-analytic reviews [17–21], it attempts to understand the mechanisms and the efficaciousness of a psychological ‘talking’ therapy in a population of individuals with severe mental health problems who are highly vulnerable to suicidal thoughts and acts. A more complex trial design is inappropriate given that the CARMS RCT advances pilot work.

The primary outcome is suicidal thoughts and acts at 6 months following entry to the trial. It is predicted that i. suicidal thoughts and acts will be less frequent and less severe in the intervention condition compared to the control condition, measured at therapy cessation (6 months) and after a 12 month follow up (FU) period compared to baseline; ii. negative appraisals of social support, emotional regulation, and interpersonal problem solving will lead to stronger perceptions of being defeated, trapped and hopeless, which will in turn lead to suicidal thoughts and behaviours over time; and iii. the treatment condition will result in less severe negative appraisals, and reduced perceptions of defeat, entrapment and hopelessness compared to the control condition. A more exploratory prediction is that symptoms of psychosis, particularly positive symptoms of hallucinations and delusions and their associated distress, will strengthen the relationships between negative appraisals, perceptions of defeat, entrapment and hopelessness and suicidal thoughts and behaviours. The acceptability and cost-effectiveness of the intervention will also be examined. Regarding the CBSPp therapy, we will attempt to gain information about the therapy process, the therapeutic alliance, engagement and adherence.

## Methods/Design

### Patient and public involvement (PPI)

A central, and fundamentally important component of the CARMS RCT is that people who are Experts-By-Experience (EBEs) have been involved in the design and set up of this project. Indeed, some of the co-investigators on the CARMS trial have personal experience of suicidality. We have established a CARMS specific PPI group with suicidal experiences and severe mental health problems who will be invited to have direct involvement in all the research stages, decision making processes, and suicidality training processes, of the CARMS project throughout its duration. It is becoming increasingly recognized that this type of PPI involvement is essential in any research projects investigating mental and physical health issues including those with economic implications [45–48].

### Study design: the RCT

The CARMS trial involves both an RCT and a nested qualitative component within the design. The trial was registered with ClinicalTrials.gov (reference NCT03114917) where it was first posted 14th April 2017. It was also registered with ISRCTN (reference ISRCTN17776666 10.1186/ISRCTN17776666) with registration being assigned 5th June 2017). Registration was accepted before recruitment commenced. The design of the trial follows Consolidated Standards of Reporting Trials (CONSORT; http://www.equator-network.org/reporting-guidelines/consort/) and SPIRIT guidelines (Standard Protocol Items: Recommendations for Intervention Trials; http://www.spirit-statement.org/), and the TIDieR checklist and guide; Template for Intervention Description and Replication (*BMJ* 2014; 348:g1687).

**Fig. 1:**
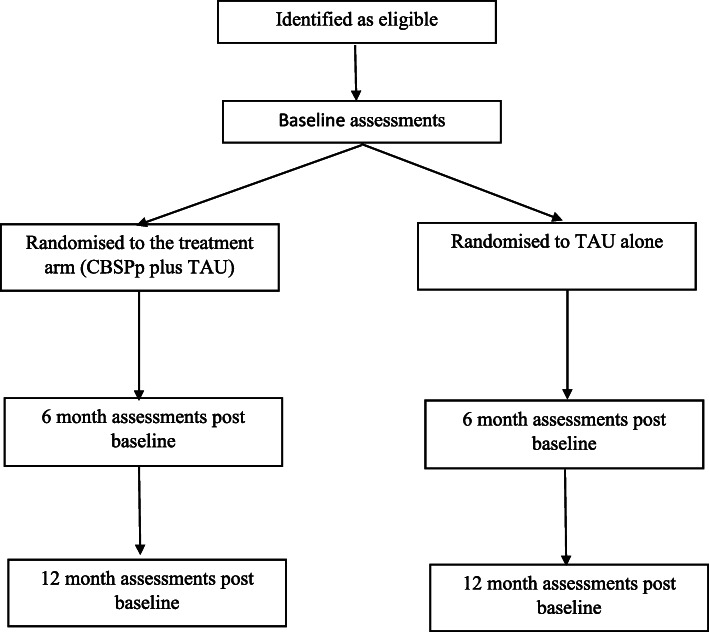
The CARMS Trial design

The design is a single-blind RCT with two parallel arms. Outcome variables assessing suicidal experiences will be collected at baseline, and at 6 and 12 months follow-up time points. Data pertaining to clinical and mediational variables will be collected at baseline, after therapy cessation (6 months), and at the 12 month follow-up time point. Health economics measures (assessed over the previous 6 months) will be collected at baseline and at the 12 month follow-up time-point to allow comparison over the pre-trial and post randomization period. It is anticipated that up to 24 individual therapy sessions, of up to an hour, will be offered over 6 months. Participants will be independently randomised to one of the two trial arms, with stratification based on whether the participant is or is not prescribed anti-depressant medication (as this could affect the primary outcome variable) and the NHS sites over which the trial is being tested (see Fig. 1).

### Study design: the qualitative component

There are four qualitative work-streams. The aim of the first is to investigate perceptions of both participants and health professionals, including mental health professionals, concerning aspects of the implementation of a psychological talking therapy focused on ameliorating suicidal thoughts and behaviours in people experiencing non-affective psychosis. The second work-stream explores psychological mechanisms which are perceived to be precursors and drivers to suicidal thoughts and acts. The third, examines acceptability of the experience of the therapy from the perspective of those randomised to CBSPp. The fourth work-stream investigates the experiences of participants taking part in research which has a suicide focus.

A purposive sampling matrix will be used to maximise diversity in the participants recruited to the qualitative work streams. Semi-structured, one-to-one, interviews will be used across all the qualitative work streams to generate the data.

### Recruitment to the RCT (the host site is Greater Manchester Mental Health NHS Foundation Trust)

Potential participants will be identified by mental health professionals in the participant’s mental health care team (including community mental health teams, early intervention services, and inpatient psychiatric wards) using the inclusion and exclusion criteria listed below. These professionals will then ascertain whether participants are willing to receive information about the study and to be contacted by a CARMS researcher. If so, potential participants will be contacted in order to provide them with a Participant Information Sheet (PIS) and to check eligibility for the trial. At least 24 h after receiving the PIS, participants will be asked to confirm whether they want to take part in the CARMS project and, if so, to provide informed consent.

Posters and flyers will be distributed in areas accessible to potential participants e.g., health service waiting rooms, inpatient wards, relevant community venues, where feasible. In addition, information about the CARMS trial will be disseminated via mental health charities. Recruitment materials will be used to enable participants to request that a member of their mental health care team (e.g., care co-ordinator) refer them into the CARMS trial. A dedicated CARMS website will also be used to provide information about the CARMS trial (https://sites.manchester.ac.uk/carms/). In addition, recruitment materials (e.g., posters, flyers, newsletters) will be distributed to mental health service providers. Members of the CARMS team, including researchers and therapists, will deliver mental health team briefings about CARMS as appropriate.

### Study design: eligibility criteria

The eligibility criteria are i. ICD-10 diagnosis relating to non-affective psychosis (ICD 10, i.e., F20 – F29), ii. self- reported experiences of suicidal thoughts, urges, plans and/or acts in the past 3 months, iii. in contact with mental health services and under the care of a mental health services clinical team (e.g., NHS community or inpatient mental health care teams, early intervention teams, crisis teams, home treatment teams), iv. aged 18 or over, v. sufficient English to complete questionnaires (hence, not needing an interpreter), vi. able to give informed consent as assessed by either a responsible clinician or by CARMS research staff following the British Psychological Society’s guidelines on gaining informed consent (http://www.bps.org.uk/sites/default/files/documents/code_of_human_research_ethics.pdf).

The exclusion criteria are i. dementia, or an organic brain disorder, ii. unable to complete assessments due to language barriers, and iii. currently taking part in a clinical trial. A short screening procedure will be used to assess eligibility.

### Study design: withdrawal criteria

The withdrawal criteria are i. the participant decides to withdraw from the trial for any or no reason, and ii. the participant is lost to follow-up. However, data will be included in the analyses up to the point of withdrawal with appropriate consent.

### Study design: randomisation and blinding

Randomisation will take place only when participants have consented to participate and after the baseline assessments have been completed. Hence, participants will consent to take part without knowing their group allocation. The Manchester Academic Health Sciences Centre (MAHSC), Clinical Trials Unit (CTU) will oversee the blocked stratified randomisation procedures. Researchers conducting the assessments, the trial statistician, one of the CARMS Trial Co-Principal Investigators, and members of the CTU conducting data handling and data monitoring will be blind to the randomised allocation group of participants. Procedures will be in place if a CARMS researcher becomes unblinded. In such an instance of unblinding then a different researcher will be allocated to work with that participant. We will not disclose the reason for this allocation. Indeed, we will strategically re-allocate researcher resource so that re-allocation is a frequent occurrence for researchers, especially given the wide geographical distribution of sites.

### Study design: sample size and power calculations

Based on power analyses, the target sample size is 125 participants in each arm (treatment and control), meaning that there will be a target of 250 participants in total for the primary analyses at 6 months across the participating sites. To account for attrition of 25%, it is anticipated that up to 333 participants will be recruited into the baseline phase of the trial. It is estimated that 50% of people approached will decline to take part. Therefore, approximately 666 participants will be identified as eligible and screened.

We will use an approach based on a simple t-test for the between group comparison in the primary suicide outcome measure which is specifically designed to account for differential clustering or partial nesting between the two arms [49]. It is implemented using the command clsampsi- in Stata [50]. This approach requires the following assumptions:

- Effect size: A clinically meaningful difference on the primary suicide outcome variable (ASIQ) from baseline to the 6 month follow-up time-point is estimated as a 16 point reduction, which corresponds to an effect size of 0.42, based on pilot data [30].
- Attrition: We are allowing for an attrition rate of 25% from baseline to final follow-up.
- Clustering: we account for clustering of outcomes within the participants sharing a therapist in the treatment arm with an ICC = 0.02.
- Random allocation: 1:1 random allocation, 0.05 significance level, and 80% statistical power.

For our proposed mediational analyses to test psychological mechanisms, a sample size of approximately 250 has greater than 80% power to detect a proportion mediated of 35%, and over 70% power to detect a proportion mediated of 30% (calculated using PowerMediation in R).

In sum, our sample size will have sufficient power for our proposed efficacy and mediation analyses.

### Study design: study arms of treatment and control groups

#### Treatment arm: the CBSPp intervention plus TAU anticipated as 24 weekly 50 min sessions

The CBSPp intervention is a one-to-one, formulation based approach. Initial sessions focus on engaging the individual in exploring their suicidal thinking and behaviours and how they relate to key underlying psychological constructs and to their key emotions, cognitions and behaviours. Ways in which these types of thoughts and behaviours interact with their experiences of psychosis, life circumstances and history is also explored to reach a shared formulation and understanding of their suicidal thoughts, plans, and acts. The approach, collaboratively, generates possible actions which are likely to modify negative appraisals of emotional regulation, improve perceptions of social support, and perceptions of interpersonal problem solving, and to influence other main drivers of their suicidal thoughts and acts, for example, distressing symptoms of psychosis. As a consequence, it is hypothesised that perceptions of defeat, entrapment, and hopelessness will be improved indirectly, although, perceptions of defeat, entrapment and hopelessness will also be worked on directly during the therapy [14, 30, 31]. Therapists will be trained in CBT techniques and further trained in CBSPp.

#### Control arm: TAU only

TAU will include usual clinical care. This is likely to be, although not exclusively, in the form of assessment and intervention from a multi-disciplinary mental health team as an outpatient or inpatient, prescription of psychiatric medication, regular monitoring, and social support. Where possible, we will document the types of TAU offered to and/or received by participants.

#### Proposed outcome measures: primary, secondary, mechanistic, clinical, therapy process, and health economics variables

##### Primary suicide outcome measure

This is the *Adult Suicidal Ideation Questionnaire* [ASIQ] [51] which is a self-report measure comprising 25 items. Suicide ideation is a predictor of suicide attempts and suicide death [52]. Participants are asked to report the frequency of thoughts about death and suicide in the last month using a 7 point Likert scale. For individuals attending psychiatric outpatient clinics with a history of suicide attempts, internal consistency was reported as .97. Test-retest reliability was also reported to be high (r = .95) in a mixed sample including those with a history of suicide attempts [51]. The primary analyses examine changes in the ASIQ from baseline to the 6 month time-point.

##### Secondary suicide outcome measures

We will use the following measures, the first two of which are self-report measures.

1. *The Suicide Probability Scale* [53] comprises 36 items and measures four components of suicidal experiences including suicidal ideation, hopelessness, negative self-evaluations and hostility. The internal reliability for this measure was found to be high in a sample including psychiatric inpatients with a Cronbach’s alpha score reported as .93 [53].
2. The *Beck Scale for Suicidal ideation* [54] measures recent suicidal ideation, plans, and intent over the past week (19 items) together with previous attempt history (2 items). This self-report measure has been reported as having an alpha coefficient of 0.96 and test–retest reliability of r = 0.88 with people who were psychiatric inpatients and with those with experiences of non-affective psychosis [55].
3. *Self-reported frequency of suicidal thoughts, plans and attempts over the past 6 months.* Where feasible, we will collect data from clinical case notes and documentation of adverse events, including those considered to be serious.

##### Mechanistic outcome variables

Each mechanistic variable assesses key components of the predicted psychological pathways to suicidal experiences [22] using self-report questionnaires:

1. The *Difficulties in Emotional Regulation Scale* [56] measures self-reported appraisals of emotional control. Four aspects of emotional regulation comprise the scale which are i. an awareness and reflective capacity, or understanding, with respect to emotions, ii. the ability to accept thoughts and behaviours reflective of emotions, iii. feeling in control of emotional reactions, and iv. feeling able to use effective emotional regulation strategies. Typically, total scores on this scale have been found to correlate positively with a range of measures of psychological mental health problems in a large sample presenting at an outpatient psychiatric clinic. In addition, internal reliability was reported as 0.97 in this sample [57].
2. The *Social Problem-Solving Inventory Short Form* [58] tests appraisals of social problem solving and has five sub-scales (positive problem orientation, negative problem orientation, rational, impulsive, and avoidant problem solving styles). Overall, there are 25 items. Alpha reliabilities have been reported as ranging between 0.85 and 0.96 in adolescents, young adults, middle aged and elderly adults [58].
3. The *Social Support Appraisals Scale* [59] assesses perceptions of available social support using 23 items. Data generated from five heterogeneous student/college samples and five community samples indicated Cronbach’s alpha reliability calculations to be between 0.80 and 0.90 [59].
4. The *Beck Hopelessness Scale* [60] measures perceptions of having a negative future with 20, binary choice, yes/no responses. Hopelessness has been shown to be a robust predictor of suicidal experiences even compared with measures of depressed mood states [61]. Reliability estimates have been recorded as exceeding 0.88 in a range of samples including those with suicidal thoughts and behaviours and severe mental health problems [60, 62, 63].
5. The *Defeat and Entrapment scales* [64] assess perceptions of being defeated and trapped both of which use 16 items. Perceptions of both defeat and entrapment have been robustly implicated in pathways to suicidal experiences in people experiencing depression, anxiety [32], and also in those with non-affective psychosis [28]. In people with non-affective psychosis who also experienced some level of suicidal thoughts and behaviours, in the past or currently, the Cronbach’s alpha coefficients were reported to be 0.86 for defeat and 0.95 for entrapment [28].

##### Clinical variables

Mental health problems pertaining to psychosis, depression, and functioning, are measured in clinical interviews as follows: *Positive and Negative Syndrome Scale [PANSS]* [65], the *Psychotic Symptom Rating Scales [PSYRATS]* [66], the *Personal and Social Performance Scale [PSP]* [67] *and the Calgary Depression Scale [CDS]* [68]. The PANSS is the most widely used measure of positive, negative and general symptoms experienced by people with psychosis and was shown to have sound psychometric properties using a response analysis [69]. The PSYRATS shows excellent reliability across raters and has robust validity [66]. The PSP has excellent face validity and reliability with the majority of ratings taking place in those with schizophrenia [67]. The CDS was specifically designed for people with schizophrenia and was reported as having excellent reliability estimates [68].

Information about current medication for mental health problems (e.g., anti-psychotic and anti-depressant medication) will be recorded from self-reports and supplemented from clinical records where possible.

##### Health economics measures

Participant-level costs will be generated for each participant in both the CBSPp treatment and TAU control arms. Costs will comprise the costs of the intervention, of TAU, and downstream costs. Costs will be constructed from a combination of trial-based resource use (NHS contacts and the Client Service Use Receipt Inventory [CSRI] amended for our trial, to monitor service use) with published unit costs, allowing comparison of CBSPp with TAU in terms of costs to the NHS and PSS. Several methods are available to collect resource-use. The most popular have been resource-use questionnaires, resource-use diaries, and electronic record searches [70–72]. The Clients Service Receipt Inventory (CSRI) [73] is a commonly used method for the retrospective collection of resource-use information which has been implemented in the economic evaluation of a variety of physical and mental health care interventions [74–77]. The CSRI covers a range of economic factors including participant’s use of health and social care services, accommodation and living situation, income, employment and benefits (the CSRI is available from the Database of Instruments for Resource-use Measurement [DIRUM] website: http://www.dirum.org/assets/downloads/634462388066137028-CSRI.pdf). Similarly to other studies, we are using a modified version of the CSRI, based on the format and questions of the CSRI but ‘modified’ to reflect the care pathways of our study participants. Examples include the use of mental health care services (community teams, crisis teams, inpatient psychiatric admission),

The EQ–5D-3 L can reflect different levels of psychosis symptomatology, be responsive to intervention effects [78–80], and correlates with the PANSS and Brief Psychiatric Rating Scale - Expanded (BPRS-E) [81]. However, the EQ-5D-3 L showed significant ceiling effects compared with PANSS and BPRS-E. The EQ-5D-5 L [82] is less prone to ceiling effects in a range of health problems [83–86] and the increased sensitivity of EQ-5D-5 L may also favour Quality-Adjusted Life-Years (QALY) gains even if the changes in utility are smaller [87]. Hence we will use will use the EQ-5D-5 L. QALYs will be calculated by attaching available utility weights to the health states generated from the EQ-5D-5 L, using area under the curve methods with an assumption of a linear change between time points, controlling for baseline.

##### Demographic information

We will collect demographic information at baseline and then check at follow-up whether factors such as relationship status, work status and education have changed at the two follow-up periods of 6 and 12 months. We will collect data concerning age, ethnicity, gender, type of work, length of time doing this work, and highest level of education.

##### Assessment of mood

A visual analogue scale (0–100) will be used to assess mood prior to all assessment sessions, and after those sessions where possible. This is to get a very quick rating of whether the sessions have negatively impacted mood and will be used to feed into assessments of suicide risk.

##### Therapy process measures of therapeutic alliance, engagement and adherence

For those in the therapy arm of the trial, the therapeutic alliance will be assessed twice, after approximately four sessions and towards the end of the therapy, with the *Working Alliance Inventory – short form* [88] completed by the therapist and the participant. Therapists will also record the following information for each participant in the therapy arm of the trial as appropriate and where possible: i. number of sessions attended and ii. duration of each session.

### Data analyses of the CARMS RCT

#### Analysis of the trial data to assess efficacy

A statistical analysis plan will be prepared before any analysis is undertaken, and agreed with the independent DMEC and TSC. Reporting will follow the CONSORT for Psychological and Social Interventions. All analyses and summary statistics will be conducted on the Intention-To-Treat (ITT) population which is defined as all participants randomised regardless of completion of therapy or withdrawal from the study. All analyses will be carried out at the end of the last follow-up assessments, and no interim analysis is planned.

Consideration will be given to potential biases arising from loss to follow-up.

Analyses will be conducted in Stata version 16 or later. Descriptive statistics within each randomised group will be presented for baseline values. These will include counts and percentages for binary and categorical variables, and means and standard deviations, or medians with lower and upper quartiles, for continuous variables, along with minimum and maximum values and counts of missing values. There will be no tests of statistical significance or confidence intervals for differences between randomised groups on any baseline variable.

Treatment effects on primary and secondary outcomes will be estimated using linear mixed models fitted to outcome variables at all time points. Fixed effects will be NHS site, anti-depressant use at baseline (yes/no), baseline assessment for the outcome under investigation, treatment, time and time*treatment interactions. Participant will be included as a random intercept to account for repeated measures. If the number of therapists is different from the number of NHS sites, we will include therapist as an additional random effect.

Marginal treatment effects will be estimated for primary outcome (ASIQ score at 6 months), and for primary and secondary outcomes at all other time points, and reported separately as adjusted mean differences in scores between the groups with 95% confidence intervals and 2-sided *p*-values. Cohen’s D effect sizes will be calculated as the adjusted mean difference of the outcome divided by the sample standard deviation of the outcome at baseline. These will be displayed in a forest plot showing the treatment effects on the primary and the secondary outcomes at 6 months.

We will allow for the presence of missing data under the assumption that the data are Missing At Random, conditional on the covariates in the model, and as a sensitivity analysis include other baseline measures as predictors of future loss to follow-up.

To account for the possible prognostic effect of anti-depressant medication on outcomes, we will include anti-depressant use at baseline (yes/no) as a stratifying factor. We acknowledge that use of anti-depressant medication after randomisation might account for a proportion of any observed treatment effect because it might lie on the causal pathway between randomisation and outcome but it is not targeted by the intervention itself. If there is a significant differential effect in the uptake of anti-depressant medication between the treatment and control groups, we will assess the role of anti-depressant medication as a mediator, in addition to our hypothesised target mediators.

#### Analysis of the trial data to assess mechanisms

We will perform this analysis using methods similar to those of Baron and Kenny [89] but advanced by newer approaches of structural equation models, instrumental variable analyses, and principal stratification to allow for hidden confounder variables [90–92]. Moderator analyses will focus on examining the effects of psychotic symptoms as an amplifier [93]. Stata version 16 or later will be used for all the analyses.

#### Analyses of the qualitative work streams

It is anticipated that data will be largely analysed using an inductive Thematic Analysis (TA) [94, 95] approach taking an interpretative stance. Coding will be undertaken inductively at the manifest level. Following familiarisation, a coding framework will be developed and codes assigned to themes. Data generation and analysis will occur in parallel using a constant comparative approach [96]. Disconfirming evidence will be sought and the analysis refined accordingly where possible. Data generation will cease when theoretical saturation appears to have been achieved. Regular discussion of emerging codes and themes will take place with the wider research team which includes service users, clinical and academic psychologists, and psychiatrists. This is a recognised method for maximising the trustworthiness of the final analysis [97].

#### Economic evaluation

Patient-level costs will be generated for each participant in the treatment (CBSPp intervention plus TAU) and TAU alone arms of the trial. Costs will comprise those of the treatment arm, those of TAU arm, and downstream costs. Costs will be constructed from a combination of trial-based resource use (NHS contacts and the Client Service Use Receipt Inventory [CSRI] amended for our proposed trial, to monitor service use) with published unit costs, allowing comparison of treatment with TAU in terms of costs to the NHS and PSS. The CSRI [98] is a commonly used method for the retrospective collection of resource-use information which has been implemented in the economic evaluation of a variety of physical and mental health care interventions [75, 76, 99, 100]. The CSRI covers a range of economic factors including a participant’s use of health and social care services, accommodation and living situation, income, employment and benefits (CSRI is available from the Database of Instruments for Resource-use Measurement (DIRUM) website: (http://www.dirum.org/assets/downloads/634462388066137028-CSRI.pdf). Similarly to other studies, we are using a modified version of the CSRI, based on the format and questions of the CSRI but ‘modified’ to reflect the care pathway of our study participants.

Participant-level costs will be generated for each participant in the Treatment and TAU arms from a combination of trial-based resource use with published unit costs. The unit costs of resource use will be taken from publicly available sources including current editions of NHS reference costs and the Unit Costs of Health & Social Care [101, 102]. Costs will be compared between the two groups using a bootstrapped regression model (as the data are likely to be skewed).

#### Custodians of the data

It should be noted that the Co-Principal Investigators are the custodians of the data.

## Discussion

The risk of suicide fatalities in people who experience severe mental health problems, including those with non-affective psychosis, is extensively elevated compared to suicide fatalities in the general population [8, 10, 103]. In addition, the distress of experiencing suicidal thoughts and acts can be severe, perhaps, especially when linked with mental health problems, such as, hallucinations and delusions [9, 104–106]. Within the existing literature, there has been a tendency to focus on the extent to which therapies, including psychological talking therapies, result in the reduction of symptoms indicative of mental health problems rather than on redressing suicidal experiences [14, 30, 31, 107]. Evidence indicates that suicidal experiences need to be targeted by psychological talking therapies directly [18, 30, 31, 108]. However, the development of talking therapies for suicidal experiences is in its infancy [14, 30, 31, 107, 109]. The CARMS project, i.e., a multifaceted RCT, is important because it redresses this gap.

Six strengths of the CARMS RCT should be emphasised, and discussed.

First, the therapy being evaluated in the CARMS RCT is based on a theoretical psychological model, namely the SAMS, of the psychological pathways thought to underlie suicidal experiences [22]. The SAMS resonates with other recent psychological approaches to understanding suicidal thoughts and acts [23–26]. Both the SAMS, and contemporary psychological models of suicidal experiences, have gained support from qualitative, quantitative and epidemiological methods [22, 28, 32–34, 63, 110–119].

Second, the CARMS CBSPp intervention has been developed from evidence based on working with people with a range of severe mental health problems, such as those given diagnoses of schizophrenia, PTSD, the bipolar disorders and depression [32, 62, 63, 107, 111–115, 120]. This means that the intervention has a mechanistic, scientific basis which examines both transdiagnostic and the specificities of mental health problems, for example hallucinations and delusions, in the pathways to suicidal thoughts and behaviours [9]. In accord with this principle, the CARMS trial aims to examine the extent to which specific experiences of psychosis, such as, the distress caused by hallucinations and delusions are primary triggers for suicidal experiences and/or amplify other known psychological precipitants of suicidal thoughts and experiences, such as, a sense of hopelessness, defeat and entrapment.

The third strength is that the psychological talking therapy, i.e., CBSPp, used in the CARMS trial is suicide focussed, personalised, and formulation driven. This psychological talking therapy is supported by evidence pertaining to acceptability, feasibility and efficaciousness from qualitative and quantitatively designed pilot work with people living in the community with psychosis, prisoners, and psychiatric in-patients [30, 31, 43, 107–109, 121–123]. These individuals are exceptionally vulnerable to suicidal thoughts, acts and deaths because of their complex, and often severe, mental health problems.

Fourth, CARMS aims to assess not only the efficaciousness of our suicide-focussed psychological intervention, but to advance our understanding of the psychological mechanisms underlying suicidal thoughts and behaviours. It is vitally important to advance these mechanisms in a context of understanding how to address the cyclical, and often, unpredictable patterns in which people transition from, and between suicidal thoughts, urges, plans and acts [118, 119].

Fifth, there are four qualitative work streams nested within the CARMS trial. These work streams will probe psychological mechanisms; barriers and facilitators to providing the CARMS trial intervention in the ‘real-world’; implementation challenges and solutions; aspects of the therapy which were perceived as being both positive and negative; and the experiences of taking part in suicide research. It has been shown that qualitative work can often contextualise aspects of the design of trials which can remain hidden wherein aspects of interventions may, otherwise, be somewhat tacit [43, 121–126].

Sixth, Patient and Public Involvement (PPI) has been used extensively in the lead-up to this CARMS trial being funded. Furthermore, people who are Experts By Experience with respect to suicidality and severe mental health problems are integral to all stages of the trial which is vital to RCT designs [45].

Four limitations of the CARMS RCT are also worthy of discussion. First, in a meta-analysis of 18 trials of cognitive based therapy for self-harm, the extent to which the therapy was perceived as effective depended on the quality of reporting the effects of the Treatment As Usual (TAU) arm of the trial with a bias in favour of trials which did not provide enough specifics of the TAU arm [127]. In the CARMS RCT an attempt will be made to collect as much information as possible regarding treatment as usual. However, it must be acknowledged that being able to collect this type of information will be challenging for a number of reasons, including, that people may access therapies from a range of sources which may be not be well documented or effectively reported.

Second, the design is single-blinded meaning that neither the therapists nor the participants can be blind to treatment. This is, of course, a necessary limitation. The CARMS researchers carrying out the assessments, those performing data entry in the Clinical Trials Unit, the trial statistician and one Principal Co-Investigator will be blinded to treatment allocation with any unblindings noted. This lends trustworthiness to the data collected and analysed which serves to counter this limitation to an extent.

Third, the participants have experiences of non-affective psychosis and recent suicidal thoughts and behaviours. That they have recent suicidal experiences is a strength of the CARMS trial. However, a limitation is that it is unclear the extent to which the CARMS therapy, i.e., CBSPp, would need to be adapted for people experiencing other mental health problems together with suicidal experiences. So, it is uncertain whether the therapy used in the CARMS trial is, or can be, generically introduced across mental health services. That said, pilot work with male prisoners and inpatients on psychiatric wards included people with a range of mental health problems, which provides some reassurance that the CARMS CBSPp intervention can be applied to people with a diverse range of mental health problems [107, 108].

Fourth, the CARMS trial assesses mechanisms and efficaciousness. It will also examine perceptions of key stakeholders (e.g., service users, mental health service providers and mental health service commissioners) about potential implementation barriers and facilitators within the qualitative work. However, it is limited in assessing whether any benefits of CBSPp can be implemented in mental health services to effect clinical beneficial change in ‘real-life’ settings, that is, outside of an RCT. That said, participants will be recruited from routine mental health services and the therapists will be individuals seconded from, and/or with experience of, mental health services which off-sets this limitation.

In conclusion, the CARMS RCT addresses a crucially important mental health need which is to determine how to diminish suicidal thoughts, urges, plans and acts in people with severe mental health problems, with a focus on those with non-affective psychosis, who are at elevated risk from death by suicide and who experience severe distress which leads them to have suicidal thoughts, to make plans to die by suicide and to make suicide attempts. The CARMS psychological intervention, CBSPp, and the RCT is theoretically grounded and supported by a range of empirical evidence based on numerous research designs in diverse populations.

## Data Availability

The datasets used and/or analysed during the current study are available from the corresponding author on reasonable request.

## Abbreviations

ASIQ: Adult Suicidal Ideation Questionnaire
BPRS-E: Brief Psychiatric Rating Scale
BMJ: British Medical Journal
CARMS: Cognitive AppRoaches to coMbatting Suicidality
CBSP: Cognitive Behavioural Suicide Prevention
CBSPp: Cognitive Behavioural Suicide Prevention for psychosis
CBT: Cognitive Behavioural Therapy
CDS: Calgary Depression Scale
CONSORT: Consolidated Standards of Reporting Trials
CSRI: Client Service Use Receipt Inventory
CTU: Clinical Trials Unit
DMEC: Data Monitoring and Ethics Committee
DIRUM: Database of Instruments for Resource-use Measurement
EBE: Experts-By-Experience
EME: Efficacy and Mechanism Evaluation
FU: Follow Up
ICD-10: International Statistical Classification of Diseases 10th version
ISRCTN: International Standard Randomised Control Trial Number
ITT: Intention-To-Treat
MAHSC: Manchester Academic Health Sciences Centre
MRC: Medical Research Council
NHS: National Health Service
NIHR: National Institute for Health Research
PANSS: Positive and Negative Syndrome Scale
PPI: Patient and Public Involvement
PSP: Personal and Social Performance Scale
PSS: Personal Social Services
PSYRATS: Psychotic Symptom Rating Scales
QALY: Quality-Adjusted Life-Years
RCT: Randomised Controlled Trial
SAMS: Schematic Appraisal Model of Suicide
SPIRIT: Standard Protocol Items: Recommendations for Intervention Trials
TA: Thematic Analysis
TAU: Treatment As Usual
TIDieR: Template for Intervention Description and Replication
TSC: Trial Steering Committee
UK: United Kingdom
USA: United States of America
